# Diet and trophic niche of the invasive signal crayfish in the first invaded Italian stream ecosystem

**DOI:** 10.1038/s41598-021-88073-2

**Published:** 2021-04-22

**Authors:** Fabio Ercoli, Daniela Ghia, Laura Gruppuso, Gianluca Fea, Tiziano Bo, Timo J. Ruokonen

**Affiliations:** 1 Chair of Hydrobiology and Fishery, Institute of Agricultural and Environmental Sciences, Estonian University of Life Sciences, Kreutzwaldi 5D, 51006 Tartu, Estonia; 2 Department of Biological and Environmental Sciences, University of Jyväskylä, Survontie 9C, 40014 Jyväskylä, Finland; 3 Dipartimento di Scienze della Terra e dell’Ambiente, Università degli Studi di Pavia, Pavia, Italy; 4 Dipartimento di Scienze della Vita e Biologia dei Sistemi, Università degli Studi di Torino, Via Accademia Albertina 13, 10123 Turin, Italy; 5 NaturaStaff Hydrobiologist, Via Lunga, 14040 Mongardino, AT Italy; 6 Natural Resources Institute Finland, Survontie 9A, 40500 Jyväskylä, Finland

**Keywords:** Freshwater ecology, Invasive species, Stable isotope analysis

## Abstract

The occurrence of the signal crayfish *Pacifastacus leniusculus* in the Valla Stream was the first established population of this invasive species recorded in an Italian stream ecosystem. We evaluated the seasonality of diet and trophic niche of invasive signal crayfish in order to estimate the ecological role and effects on native communities of the stream ecosystem. We studied the differences in food source use between sexes, life stages and seasons using carbon and nitrogen stable isotope analyses. To supplement stable isotope analyses, we evaluated food source usage using traditional stomach content analysis. We tested the hypothesis that juveniles have a different diet, showing different trophic niches, compared to adults. Results indicated that signal crayfish adult and juvenile diets mainly rely on macroinvertebrates and periphyton in summer, shifting to mostly periphyton in autumn. Although the two age classes occupied an equivalent trophic niche, juveniles showed slightly different carbon isotope values, suggesting a somewhat ontogenetic shift consistent among seasons. No significant differences were found in adult and juvenile diets between summer and autumn seasons. Our findings suggest that signal crayfish juveniles and adults exhibited seasonal feeding habits, probably due to ecological behaviour rather than food resource availability, and that both are likely to impose similar effects on macroinvertebrate communities in this and similar stream ecosystems.

## Introduction

Signal crayfish, *Pacifastacus leniusculus* (Dana 1852), was introduced across Europe during recent decades, threatening native European crayfish and freshwater communities^1,2^. Non-native crayfish have strong negative impacts on native crayfish^3,4^, freshwater biodiversity^5–8^ and ecosystem functioning^1,9^. However, their ecological effects at different life stages, or differences between sexes, are poorly understood in natural populations. Some previous studies indicate that crayfish of all sizes feed omnivorously^10,11^, with no sexual differences^12^. However, there are also indications of ontogenetic diet shifts e.g.,^13–15^ as well as differences between males and females in predation^16^, effects on macrophyte and macroinvertebrate communities, and detritus processing^17,18^, which may result in differing trophic roles and ecological effects at different sex and life stages.^19^ found no ontogenetic shift in native population of signal crayfish, and juveniles and adults relied mostly on detrital biofilm. The diet of juvenile signal crayfish in invaded areas, and hence their contribution to effects on native communities, are poorly documented. Furthermore, crayfish impacts likely differ between sexes^16–18^ and seasons^20,21^ according to food source availability, but seasonal variation in effects on native biota have not been investigated thoroughly.

Signal crayfish is one of the most widespread invasive species in Europe, but there are few records of this species occurring in Italy. Signal crayfish were first recorded in Italy by^22^ from an Alpine river basin, successively in the Apennine region from Brugneto Lake in northwest Italy^23^, and most recently from the Valla Stream, a small tributary of the Bormida River (Piedmont, southeast Italy)^24^. To our knowledge, Valla Stream is the first Italian stream ecosystem where signal crayfish was successfully established since its first report^25^. The stream was formerly inhabited by the native, white-clawed crayfish (*Austropotamobius pallipes*), which is listed as an endangered species on the IUCN red list^26^. However, after the first record of invasive signal crayfish, the native species disappeared in the area where signal crayfish established. Signal crayfish can cause extinction of native, white-clawed crayfish^27^, which is also presumed to be the reason for local loss of native crayfish in Valla Stream. Indeed, even without carrying the crayfish plague, signal crayfish can outcompete native crayfish when coexisting e.g.^28^. Currently, white-clawed crayfish are present in headwaters of the Valla Stream tributary^29^.

In Italy, signal crayfish populations are low, and the distribution area is still rather restricted. However, signal crayfish has spread throughout other parts of Europe e.g.^2,30^, and there is little doubt that many more Italian lakes and streams will be under threat in the future. The high temperature and water scarce in summer, typical of Mediterranean streams, might influence the trophic ecology of this invasive cold-water crayfish species^31^. Signal crayfish trophic niche and diet have been already studied e.g.^32,33^ but investigations at different age classes and sex, in Mediterranean stream ecosystems are poor.

Our study aimed to assess diet and ecological roles of adult and juvenile, invasive signal crayfish in Valla Stream, Italy. We compared food source usage and trophic niches at different life stages between seasons and sexes using analysis of carbon and nitrogen isotopes and stomach contents. Based on results, we determined potential differences in signal crayfish life stages and sexes trophic roles and ecological impacts on native communities in invaded stream ecosystems. We hypothesized that diets of adult and juvenile crayfish would differ, as the life stage categories may need different proportions of nutrients for growth and maintenance. Also, we expected that diets of males and females would differ, as males grow faster than females^34^ and are more active and behaviourally dominant over females^35^, and also in relation to their reproductive status^36^. However, feeding depends on the availability of food sources; hence, the diet and ecological role will change seasonally^20,21^ based on sex and life stage.

## Results

**Figure 1:**
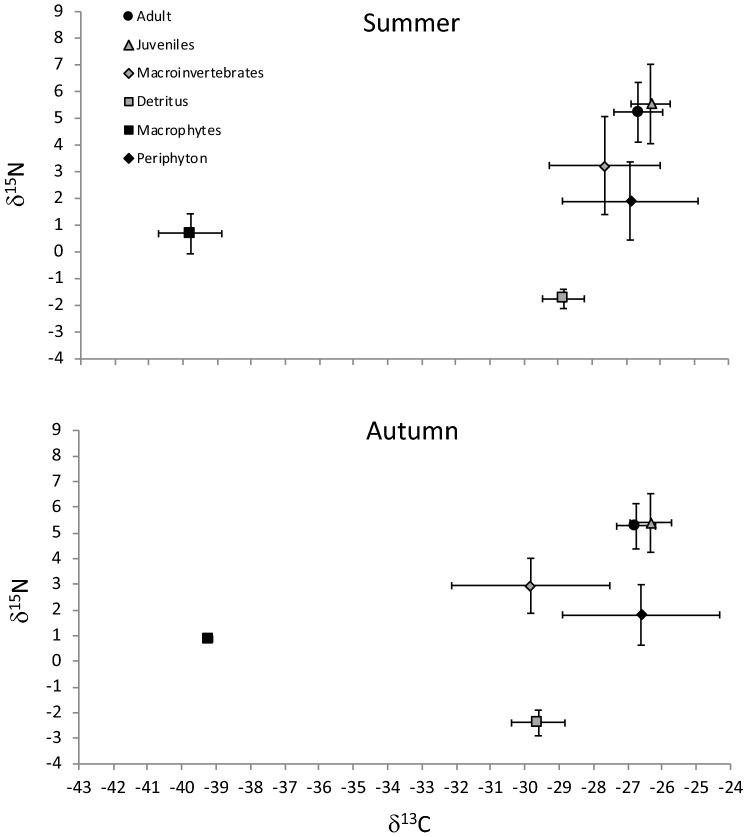
Mean (± SD) carbon and nitrogen stable isotope values (‰) of the signal crayfish size classes and of their putative food sources.

**Figure 2:**
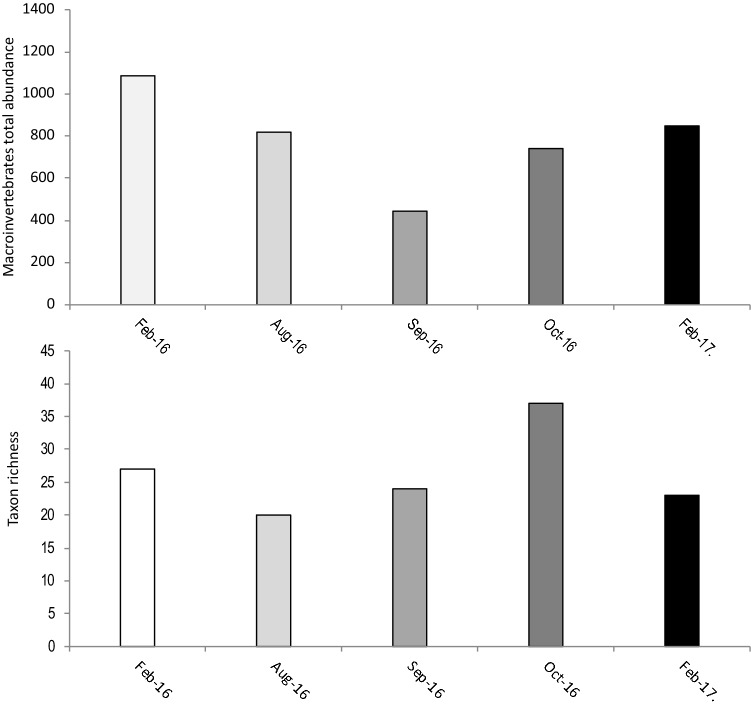
Seasons variation of macroinvertebrate total abundance and taxon richness in the upstream area adjacent to the studied site 1 of Valla Stream.

In total, 147 crayfish were collected for Stable Isotope Analysis (SIA), of which 104 were collected in summer and 43 in autumn (Table 1). Variations in nitrogen and carbon mean values for adult and juvenile crayfish were negligible among sites in summer and autumn (Table 1), though a small increase in δ^15^N values was observed at site 3 (Table 1). Carbon and nitrogen stable isotope mean values for food sources were consistent across seasons except for macroinvertebrates, which had lower mean carbon values in autumn compared to summer (Fig. 1, Table 2). Trends of macroinvertebrate total abundances were similar during the study seasons, while macroinvertebrate species richness was higher in autumn compared to summer (Fig. 2).

**Table 1:** Signal crayfish mean (± SD) stable isotope values of carbon and nitrogen, carapace length (CL), number of sampled individuals between sexes, age classes, and sites in Valla Stream in summer and autumn.

Seasons	Sites	Age class	Sex	N	CL	δ^13^C	δ^15^N
Summer		Adults	M	7	47.17 ± 14.3	− 26.38 ± 0.84	4.63 ± 0.27
			F	5	39.63 ± 10.3	− 26.53 ± 0.50	4.73 ± 0.36
	S1	Mean values			44.03 ± 13.3	− 26.44 ± 0.72	4.67 ± 0.32
		Juveniles	M	5	26.41 ± 2.6	− 26.16 ± 0.30	4.89 ± 0.12
			F	5	25.95 ± 2.6	− 26.28 ± 0.43	4.42 ± 0.21
		Mean values			26.18 ± 2.6	− 26.22 ± 0.38	4.65 ± 0.29
		Adults	M	9	49.53 ± 12.0	− 26.58 ± 0.70	4.91 ± 0.62
			F	10	47.34 ± 7.3	− 26.67 ± 0.61	5.00 ± 0.34
	S2	Mean values			48.38 ± 9.9	− 26.62 ± 0.66	4.96 ± 0.50
		Juveniles	M	8	25.39 ± 2.9	− 26.28 ± 0.43	5.03 ± 0.56
			F	12	21.58 ± 7.5	− 26.36 ± 0.56	4.95 ± 0.64
		Mean values			23.10 ± 6.4	− 26.33 ± 0.51	4.98 ± 0.61
		Adults	M	13	43.03 ± 9.0	− 27.08 ± 0.80	6.25 ± 1.60
			F	12	41.63 ± 5.2	− 26.42 ± 0.44	5.13 ± 0.99
	S3	Mean values			42.36 ± 7.5	− 26.76 ± 0.73	5.71 ± 1.46
		Juveniles	M	7	26.21 ± 2.5	− 26.53 ± 0.37	6.60 ± 1.80
			F	11	21.28 ± 8.8	− 26.10 ± 0.82	6.72 ± 1.86
		Mean values			23.20 ± 7.5	− 26.27 ± 0.71	6.67 ± 1.84
Autumn		Adults	M	3	48.97 ± 8.18	− 27.10 ± 0.88	5.03 ± 0.26
			F	3	39.38 ± 3.16	− 26.41 ± 0.21	4.69 ± 0.38
	S1	Mean values			44.18 ± 7.84	− 26.76 ± 0.72	4.86 ± 0.37
		Juveniles	M	1	26.57	− 25.95	4.36
			F	4	19.26 ± 3.78	− 25.30 ± 0.09	4.56 ± 0.43
		Mean values			20.72 ± 4.47	− 25.43 ± 0.27	4.52 ± 0.40
		Adults	M	4	37.80 ± 4.32	− 26.77 ± 0.29	4.77 ± 0.40
			F	–	–	–	–
	S2	Mean values			37.80 ± 4.32	− 26.77 ± 0.29	4.77 ± 0.40
		Juveniles	M	3	25.76 ± 1.42	− 26.38 ± 0.28	4.75 ± 0.53
			F	10	25.29 ± 2.77	− 26.86 ± 0.29	4.85 ± 0.61
		Mean values			25.40 ± 2.53	− 26.75 ± 0.34	4.82 ± 0.58
		Adults	M	4	51.38 ± 9.89	− 26.86 ± 0.70	5.97 ± 1.21
			F	2	44.24 ± 11.57	− 26.50 ± 0.11	6.05 ± 1.19
	S3	Mean values			49.00 ± 11.01	− 26.74 ± 0.53	5.99 ± 0.99
		Juveniles	M	4	24.29 ± 5.34	− 26.11 ± 0.49	6.55 ± 0.85
			F	5	22.97 ± 6.65	− 26.28 ± 0.42	6.83 ± 1.03
		Mean values			23.56 ± 6.14	− 26.21 ± 0.40	6.71 ± 0.86

**Table 2:** Mean (± SD) stable isotope values of carbon and nitrogen of food sources at each site in Valla Stream in summer and autumn.

Sites	Food sources	Summer			Autumn		
		N	δ^13^C	δ^15^N	N	δ^13^C	δ^15^N
	Macroinvertebrates	3	− 26.74 ± 1.11	3.08 ± 1.28	3	− 29.53 ± 2.33	2.31 ± 0.99
S1	Crayfish	–	− 26.34 ± 0.60	4.66 ± 0.30	–	− 26.15 ± 0.86	4.71 ± 0.41
	Detritus	3	− 28.01 ± 0.05	− 1.57 ± 0.10	3	− 30.01 ± 0.62	− 2.44 ± 0.63
	Periphyton	–	− 26.88 ± 1.99	1.91 ± 1.46	3	− 27.32 ± 0.30	0.88 ± 0.12
	Macrophytes	3	− 39.61 ± 0.20	1.92 ± 0.35	3	− 39.20 ± 0.10	0.86 ± 0.16
	Macroinvertebrates	3	− 27.92 ± 1.89	2.60 ± 1.30	3	− 30.41 ± 2.88	2.72 ± 0.82
S2	Crayfish	–	− 26.47 ± 0.60	4.97 ± 0.55	–	− 26.76 ± 0.32	4.81 ± 0.54
	Detritus	3	− 28.92 ± 0.36	− 2.07 ± 0.03	3	− 29.06 ± 0.46	− 2.62 ± 0.24
	Periphyton	–	− 26.88 ± 1.99	1.91 ± 1.46	3	− 28.24 ± 2.16	1.89 ± 1.38
	Macrophytes	3	− 40.52 ± 0.72	0.42 ± 0.17	3	− 39.59 ± 0.08	1.10 ± 0.21
	Macroinvertebrates	3	− 27.83 ± 1.34	3.95 ± 2.24	3	− 29.52 ± 1.65	3.77 ± 1.08
S3	Crayfish	–	− 26.55 ± 0.76	6.11 ± 1.69	–	− 26.42 ± 0.52	6.42 ± 0.97
	Detritus	3	− 29.18 ± 0.38	− 1.53 ± 0.31	3	− 29.76 ± 0.73	− 2.15 ± 0.31
	Periphyton	–	− 26.88 ± 1.99	1.91 ± 1.46	3	− 24.26 ± 0.74	2.65 ± 0.45
	Macrophytes	3	− 39.15 ± 0.49	0.34 ± 0.04	3	− 38.85 ± 0.03	0.85 ± 0.18

### Ontogeny and seasonality of diets and trophic niches

**Figure 3:**
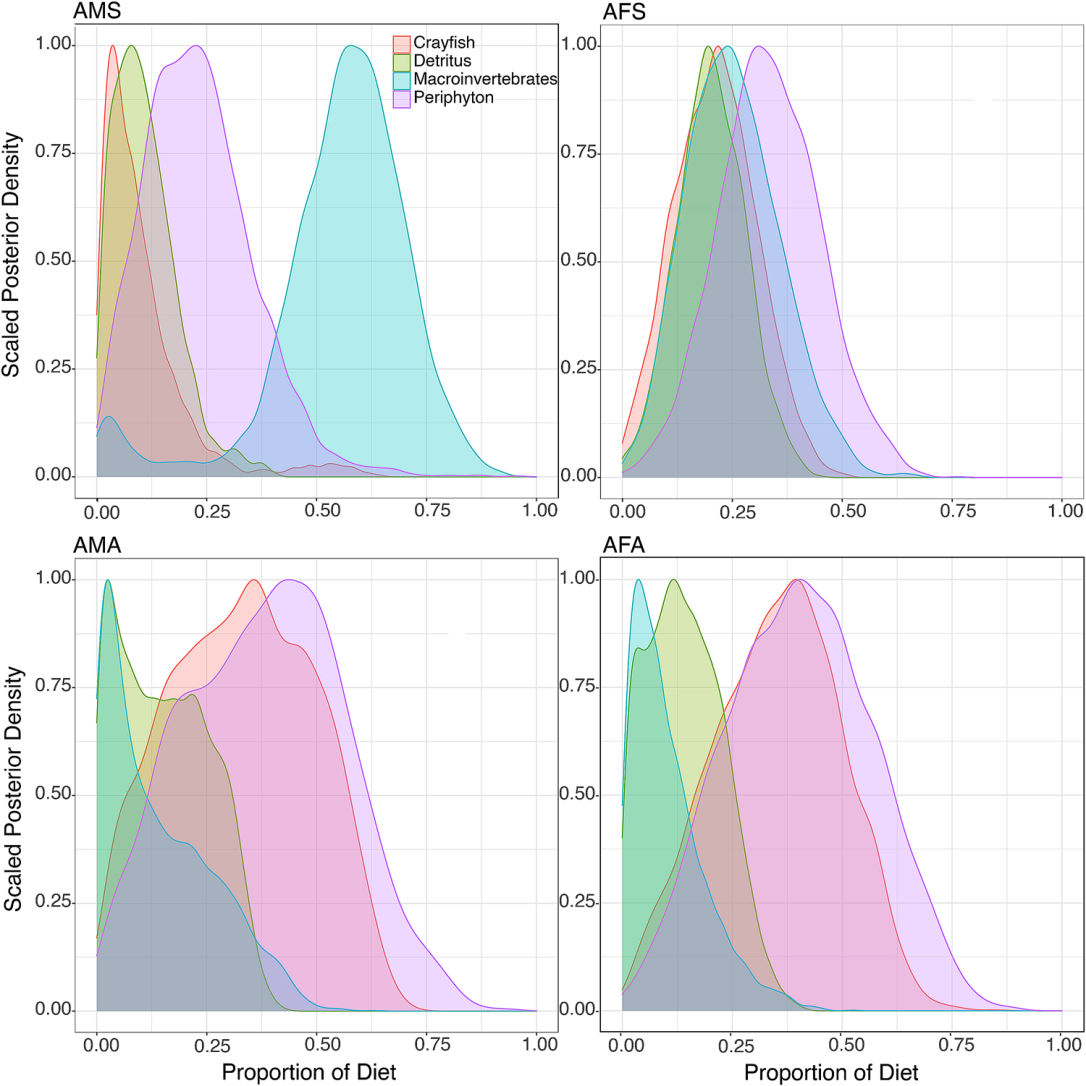
Food source proportions of adult male (AMS) and female (AFS) signal crayfish in summer, and adult male (AMA) and female (AFA) in autumn.

**Figure 4:**
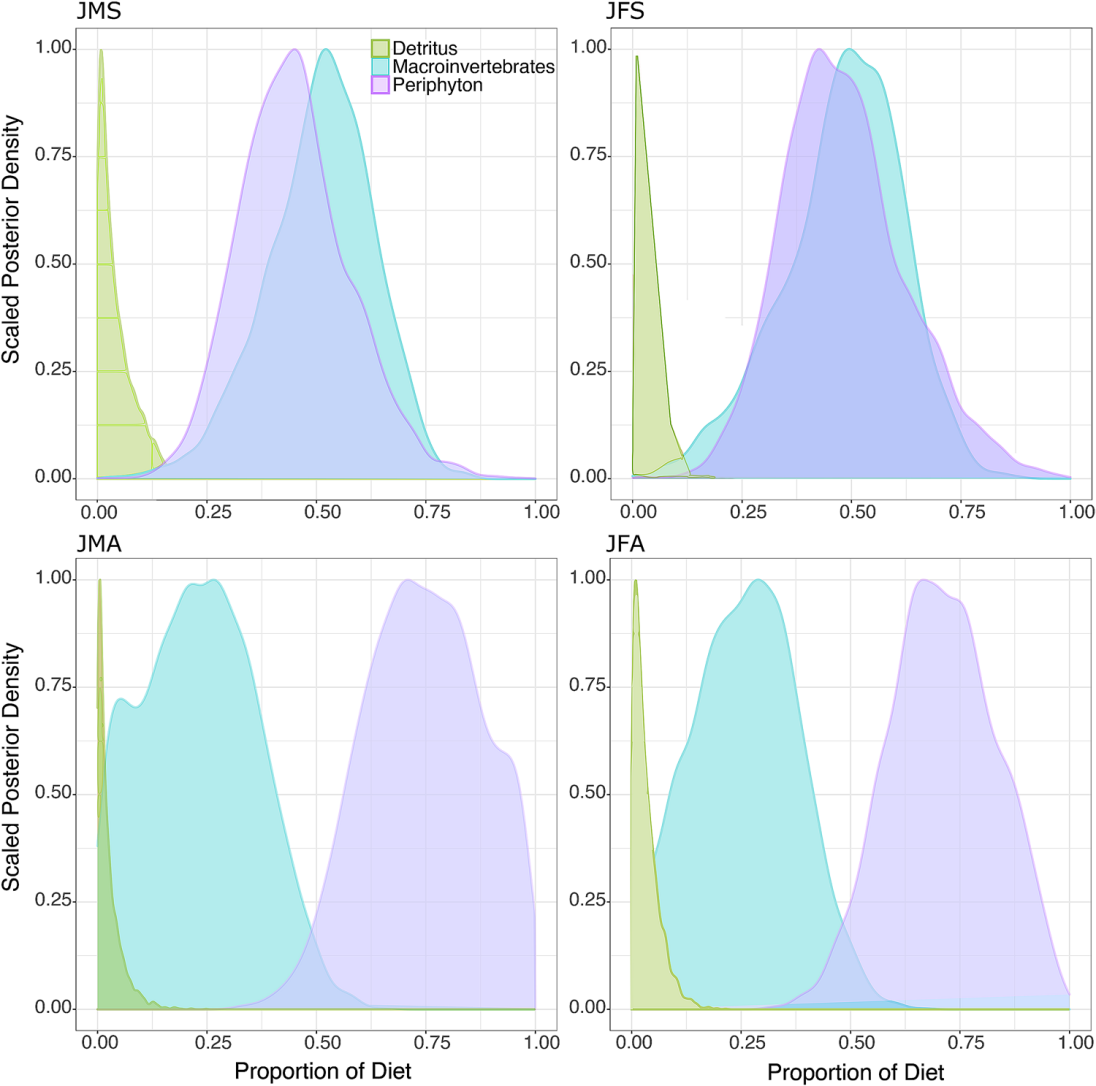
Food source proportions of juvenile male (JMS) and female (JFS) signal crayfish in summer, and juvenile male (JMA) and female (JFA) in autumn.

MixSIAR model results showed that adults and juveniles mostly relied on periphyton and macroinvertebrates during summer (Figs. 3, 4). In autumn, both life stages used mainly periphyton (Figs. 3 and 4 and Table 3). Model results indicated differences in diets of male and female juveniles between summer and autumn (Fig. 4, Table 3). Juveniles used a lower proportion of macroinvertebrates and higher proportion of periphyton in autumn. Results also indicated season-related differences in diets of adult males and females (Fig. 3, Table 3). In summer, adult males used macroinvertebrates in higher proportion, and detritus and periphyton in lower proportion, than females. Adult males used substantially more macroinvertebrates in summer than in autumn (Fig. 3, Table 3). Adult males and females showed cannibalism, with slightly higher proportions in autumn (Fig. 3, Table 3).

**Table 3:** Food source usage proportions for adult and juvenile signal crayfish in Valla Stream in summer and autumn, represented by the median diet (50% quantiles) and its 95% confidence intervals.

Seasons	Food sources	Adults						Juveniles					
		Female			Male			Female			Male		
		2.5%	50%	97.5%	2.5%	50%	97.5%	2.5%	50%	97.5%	2.5%	50%	97.5%
Summer	Crayfish	0.04	0.21	0.40	0.01	0.10	0.49	–	–	–	–	–	–
	Detritus	0.05	0.20	0.36	0.01	0.10	0.30	0.00	0.03	0.12	0.00	0.03	0.15
	Macroinvertebrates	0.07	0.25	0.48	0.04	0.58	0.80	0.17	0.49	0.72	0.25	0.52	0.72
	Periphyton	0.11	0.33	0.57	0.03	0.22	0.49	0.24	0.47	0.79	0.23	0.44	0.72
Autumn	Crayfish	0.07	0.36	0.61	0.04	0.33	0.61	–	–	–	–	–	–
	Detritus	0.01	0.14	0.31	0.01	0.15	0.34	0.00	0.02	0.11	0.00	0.02	0.11
	Macroinvertebrates	0.00	0.08	0.32	0.00	0.11	0.41	0.03	0.26	0.49	0.01	0.23	0.48
	Periphyton	0.09	0.40	0.72	0.05	0.39	0.72	0.47	0.71	0.94	0.49	0.75	0.97

**Figure 5:**
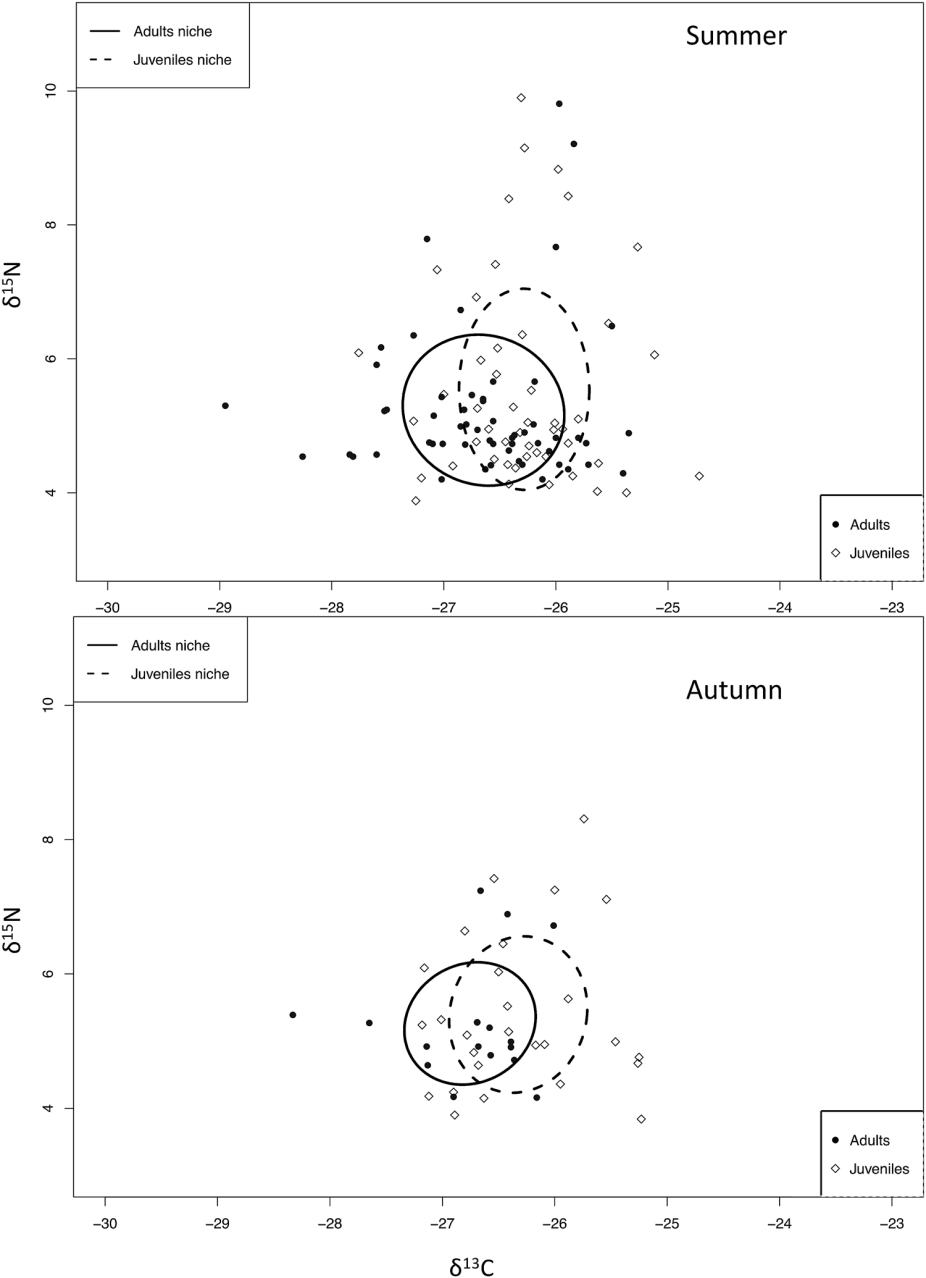
Trophic niche widths of signal crayfish adults and juveniles in summer and autumn estimated by SIBER model ellipses, which represent the feeding niche areas of signal crayfish adults and juveniles.

Trophic niche widths of adults and juveniles were similar in summer and autumn, with Standard Ellipses Area (SEAc) values of 2.31 ‰ and 2.38 ‰, respectively (Fig. 5). Furthermore, trophic niches for adults and juveniles indicated substantial overlap (0.65 and 0.62, respectively) in summer and autumn (Fig. 5).

### Stomach content analyses

**Figure 6:**
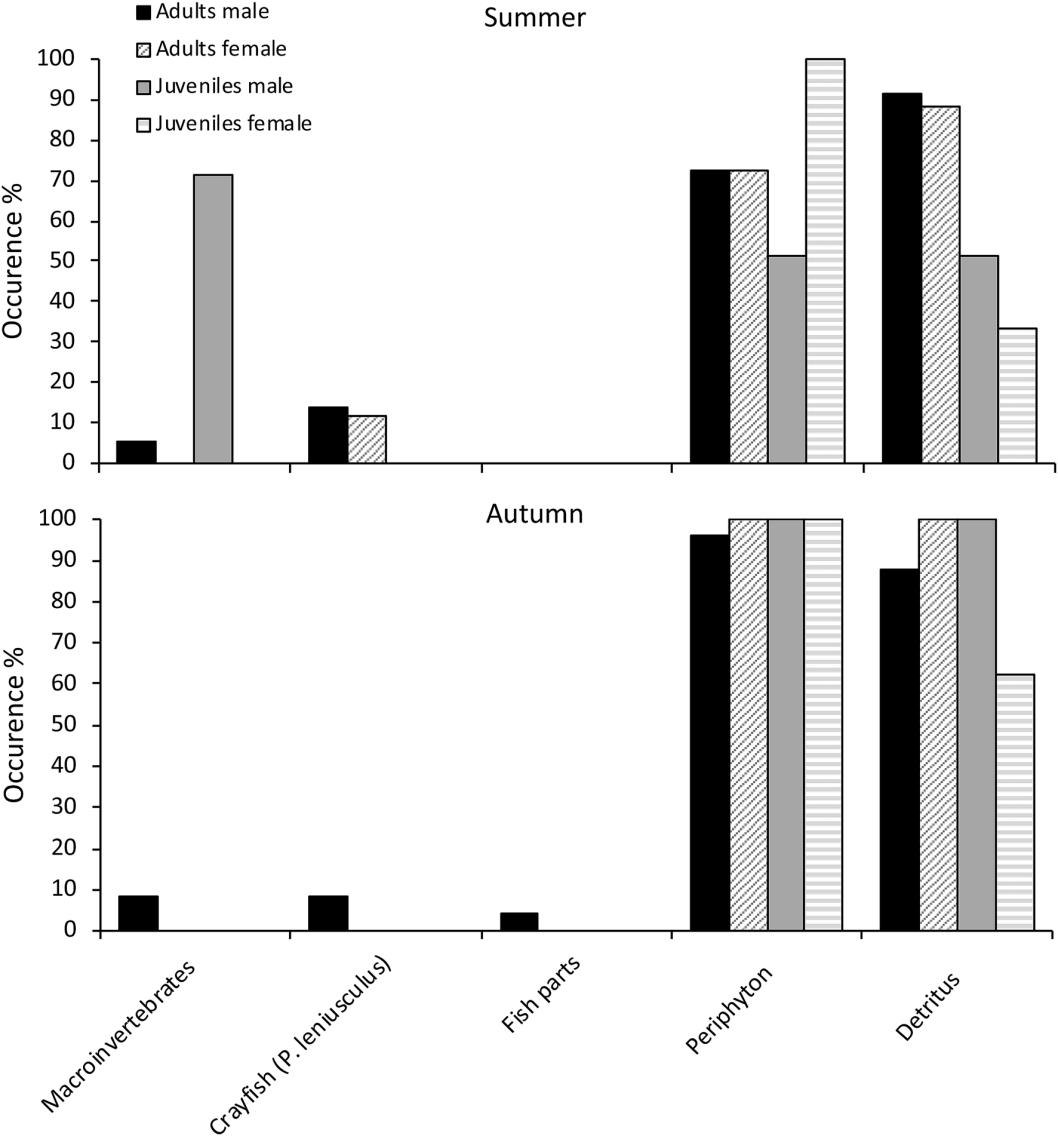
Food item occurrence in stomachs of adult and juvenile crayfish in Valla Stream in summer and autumn.

**Figure 7:**
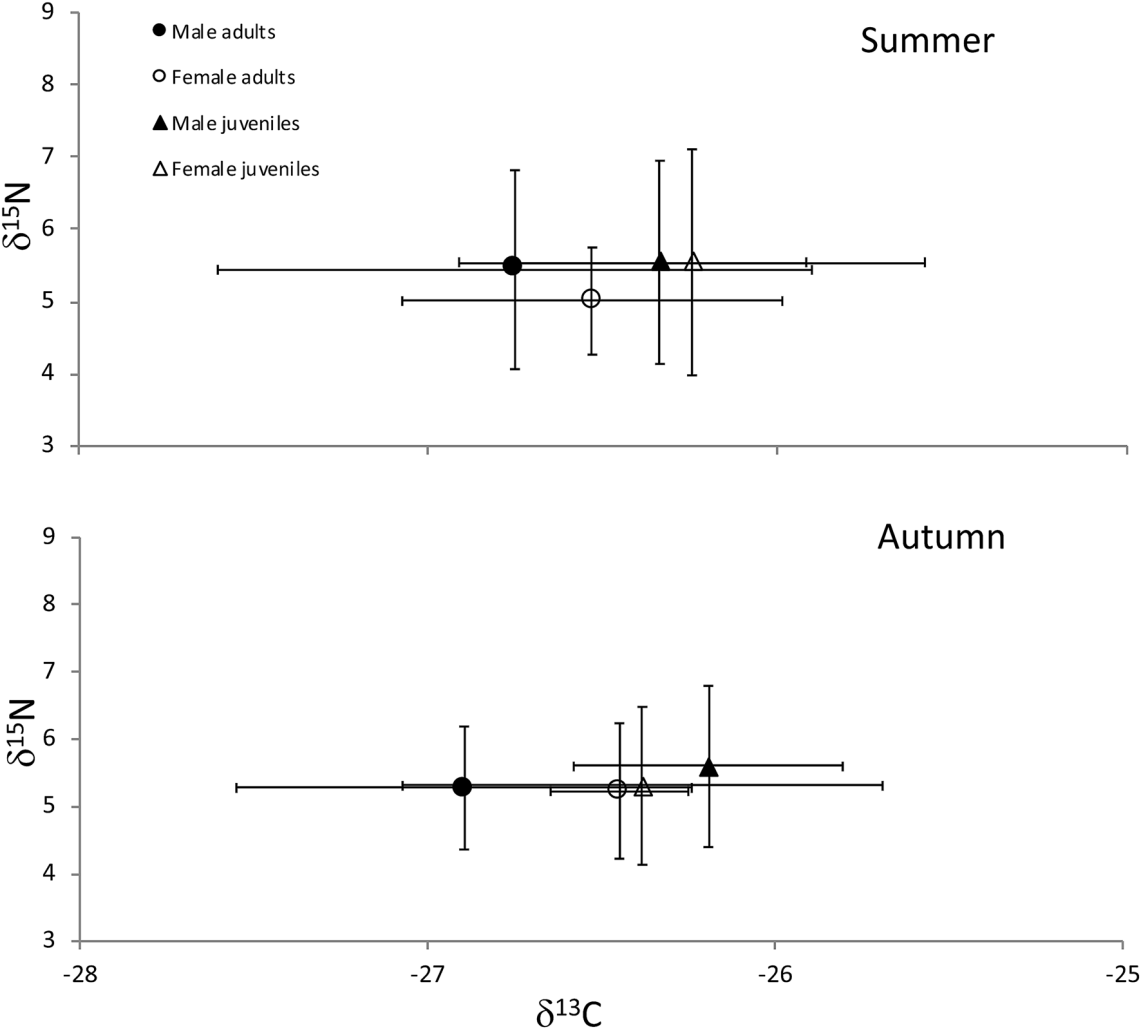
Mean carbon and nitrogen stable isotope values (‰) of signal crayfish adults and juveniles, females and males in Valla Stream in summer and autumn.

In crayfish stomachs, periphyton and detritus were the most common food sources in both seasons and for both sexes (Fig. 6). In juvenile males, periphyton and detritus were less common than macroinvertebrates, which occurred in over 70% of individuals in summer. As with stable isotope results, stomach contents in juvenile and adult males differed substantially in autumn, when adults included 9% of macroinvertebrates occurrence and juveniles 100% of periphyton and CPOM occurrence (Fig. 7). Stomach contents of juvenile females in summer and autumn consisted mainly of plant materials (Fig. 6). Although periphyton and detritus were important food sources in both seasons, they were particularly important for both life stages in autumn (Fig. 6). Adult males and females showed cannibalistic behaviour, each having similar occurrence of crayfish parts (14% and 12%) in stomachs in summer, but cannibalism was evident only in males in autumn (9%).

## Discussion

Our results showed that juvenile and adult signal crayfish relied mostly on similar food sources, which contrasts with our hypothesis of ontogenetic diet shift. In both life stages, food preferences changed seasonally, most likely due to their ecological behavior relative to temperature and biological functions rather than to seasonal food availability. Results from previous studies on ontogenetic diet shift of crayfish are inconsistent. Some showed no ontogeny^11,19,37,38^, while others found evidence of changes in diet composition between life stages^14,39^. In our study, juveniles and adults occupied the same trophic level, but the life stages had different carbon isotope values, suggesting a consistent difference in diets in both seasons. This pattern was also evident from the SIBER model, trophic niche ellipses calculation, where results indicated that, although the trophic niches overlapped, juveniles shifted slightly towards a more periphyton-based diet in autumn and summer.

Overall, stable isotope results revealed that periphyton and macroinvertebrates, and to a lesser extent detritus, represented the main food sources used by signal crayfish, while macrophytes were not included in its diet. Crayfish feed omnivorously on detritus, macrophytes, invertebrates, and vertebrates^40^. ^41^showed that crayfish generally have an animal-based diet, which supplies protein for growth, while^5^ found that plant material is an important energy source for maintenance. Thus, adult crayfish may be more carnivorous, increasing trophic level with increasing size^42^, or more detritivorous than juveniles^15,43^. In our study, both adults and juveniles ate macroinvertebrates and periphyton in summer, while periphyton was the main food source for the both life stages in autumn. This result suggests that variation in crayfish diet stems from differences in seasonal food source availability, for example due to variations in the macroinvertebrate community between warm and cold periods.^20^, in their food web study in stream, found that in general the contribution of periphyton source to consumers was greater in summer than in winter, during the low discharge period.^21^ found significant seasonal differences in detritus ate by *Orconectes sp.* which was higher in summer than in autumn, despite the more availability of leaf litter in autumn. Moreover, in line with our results, they found that periphyton source consumption was similar during both seasons, but macroinvertebrates was significantly higher in autumn than in summer. Seasonal variations in macroinvertebrate abundance and species richness were surveyed monthly (Fig. 2). Except for species richness, which increased in autumn, results showed no substantial difference between the two seasons. This suggests that variation in diet was likely due to different seasonal feeding behavior rather than food resource availability.

Crayfish are more active during warm season^44^, which was also evident in our study, as the CPUE values (Table S1—Supplementary information) were highest in summer months. Adult females and juvenile males and females are usually less active in winter, when adult females protect their eggs carried under their tails, and juveniles, still small and easily preyed upon, spend more time hiding in shelters^45,46^. This behavior is likely to avoid fish predation^47^ and to avoid aggressive and cannibalistic adults^48^. This behavior might explain less foraging activity by adult females and juveniles in autumn, as indicated by our gut contents results.

Cannibalism is common in signal crayfish adults^48^. Our results confirm this finding, although males showed cannibalistic behavior only in autumn.

Gut content results generally agreed with food source use revealed by stable isotope analysis. Our results were partially in contrast with findings of^49^, in which both life stages affected macroinvertebrates abundance, and adults consumed more detritus than juveniles. However, macroinvertebrate prey items were recorded only from stomachs of juvenile and adult males in our study, with a remarkably high percentage from juvenile males in summer. This difference between sexes could be explained by the selective consumption, where males would prefer more energetic food source respect to females^50^. The high percentage of macroinvertebrates found in juveniles in summer might be due to the fact that animal-based diet is more important for the growing of juveniles than for adults, especially in summer when juveniles growth is likely more intensive^51^. On the other hand, our results agree with^34^ and^19^, showing high percentages of plant material in stomach contents from both life classes. However, no macroinvertebrate prey were found in adult and juvenile females, which instead exhibited high occurrence of plant material in our study.

Contrary to our expectations, results did not show a clear ontogenetic shift. The lack of a distinct difference in adult and juvenile diets might be due to most juveniles being represented by individuals 1 + or 2 years old, and their diets might have already shifted towards an adult diet.^34^ also suggested that similar diets of adult and juvenile signal crayfish might be related to using 2 year old juveniles, which had already changed their diet. More investigations on ontogenetic niche shifts using young-of-the-year juveniles are needed to better understand juvenile diet shifts.

Our results revealed important findings on ontogenetic effects of signal crayfish. The combined pressures exerted by both adults and juveniles can affect the stream ecosystem at several trophic levels and threaten native macroinvertebrate communities and ecological function of the study stream. Actions are urgently needed to stop the spread of invasive signal crayfish in Italy and protect native stream ecosystems and endangered, native, white-clawed crayfish*.* Total eradication of invasive crayfish is laborious and probably an impossible task, but intensive trapping^52^, together with hand/kick-net^38^ removal of smaller crayfish, could help prevent spread to non-invaded areas and decrease negative ecological impacts of signal crayfish.

## Material and methods

### Study area

**Figure 8:**
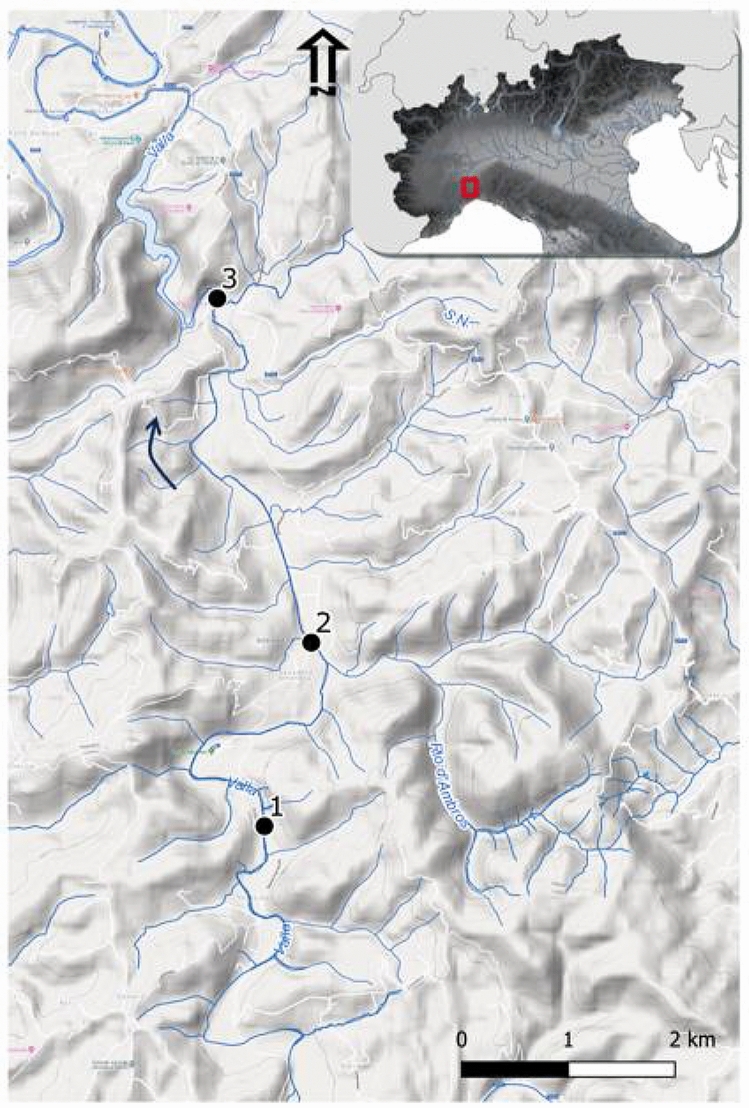
Map of Valla Stream and sampling sites. The arrow indicates the direction of flow. The base map is from Google Terrain Hybrid (https://cloud.google.com/maps-platform/terms) and the hydrography layer available by Regione Piemonte WMS service (http://www.geoportale.piemonte.it). The map was created using QGIS 3.10 LTR—A Coruña (https://www.qgis.org).

The study was conducted in Valla Stream, an Apennine stream belonging to the Po River basin and situated in northwest Italy (Fig. 8). Valla Stream flows south-north for 24 km, from 833 to 222 m a.s.l., with an average slope of 2.2%. Its lower part was dammed for hydroelectric power production in 1923–1925. The 42.5 m high dam forms a lake about 100 m wide and more than 2 km long. This stream is a typical, third-order, Apennine lotic environment of 4.0–4.5 m width. During summer, the lower part of the stream usually dries out, or isolated pools may remain. Riparian vegetation is dominated by alder (*Alnus glutinosa*), willow (*Salix* spp.), poplar (*Populus* spp.), and sporadically by black locust (*Robinia pseudoacacia*), while the surrounding land use is characterized by oak (*Quercus robur)* and hornbeam (*Carpinus* spp.) forest, field/pasture and scattered houses. The substrate is composed of coarse-grained, arenaceous, and conglomeratic successions with clayey-arenaceous strata.

### Sample collection

For our study, three sampling sites (200 m stretch) were chosen in the lower part of the stream (along an 8 km reach), for similar environmental features and substrate composition (Fig. 8). To assess signal crayfish abundance in Valla Stream, catch per unit effort (CPUE) was collected monthly from all sites using baited traps from April 2015 until March 2016 (Table S1 Supplementary information). Crayfish individuals for stable isotopes and gut content analyses were collected by hand on June 2015 and November 2016, when mean water temperatures were 25 °C and 9 °C, respectively. In this study, no specific permissions were required for animals sampling.

From each site, samples of macroinvertebrates, detritus (coarse particulate organic matter; CPOM), periphyton, and macrophytes as putative food items were collected. Three replicates of each food item were collected in summer and autumn at each sampling site. Detritus (CPOM) and periphyton represented terrestrial allochthonous (oak (*Quercus robur),* alder (*Alnus glutinosa*), willow (*Salix* spp.), poplar (*Populus* spp.) and black locust *(Robinia pseudoacacia*)) and in-stream primary production, respectively. Macroinvertebrates were sampled by kick-net, while signal crayfish specimens, macrophytes and detritus were collected by hand. Periphyton samples were collected gently by brushing stone surfaces found along the stream bed. All samples were kept cool in the field and frozen after return to the laboratory within a few hours of collection.

Periphyton was then thoroughly scanned under stereoscope for the removal of macroinvertebrates. Periphyton samples from summer were not available for stable isotope analyses. We used variation in carbon and nitrogen isotope values of periphyton between autumn and summer from previous studies in temperate streams with similar biome type, catchment area (km^2^), vegetation composition to our study stream^53^. Among these streams, those with carbon and nitrogen stable isotope range values in autumn similar to the ranges of our study sites from same season (from -23.27 to -31.29 for carbon and from 0.26 to 3.74 for nitrogen), were selected to calculate and represent periphyton summer carbon and nitrogen values in our studied stream. In addition, quantitative macroinvertebrate samples were collected using a kick-net in the upstream area adjacent to site 1, which represented the stream stretch studied from February 2016 to February 2017, to test for seasonal variability in abundance and species richness. After collection, samples were immediately preserved in 70% ethanol. In the laboratory, samples were sorted, identified to the lowest feasible taxonomic level (mostly to species or genus), and counted.

Crayfish sexes were determined, and carapace lengths (CL) were measured to the nearest mm, in order to divide individuals in two different age classes (adults ≥ 30 mm, and juveniles < 30 mm)^54^ (Table 1). A piece of untreated abdominal muscle tissue from each crayfish was used to measure the stable isotope ratios as recommended by^11^. Signal crayfish were analyzed individually for stomach contents and prepared for stable isotope analyses.

### Stable isotope analysis (SIA)

All samples for isotope analysis were oven dried for 48 h at 60 °C to constant weight and ground to a fine, homogenous powder. Animals and plant samples were then weighed (0.6 mg for animals and 1.5 m for plant material) into tin caps and encapsulated. Analyses of carbon and nitrogen stable isotopes were conducted with a FlashEA1112 elemental analyzer coupled to a Thermo Finnigan DELTAplus Advantage continuous flow isotope ratio mass spectrometer (Thermo Electron Corporation, Waltham, MA, USA) at Jyväskylä University in Finland. Stable isotope values of carbon and nitrogen are expressed in delta notation as parts per thousand (‰) according to:

1 $$\delta X = (Rsample / Rstandard - 1) \times1000$$

where X is either carbon or nitrogen isotopes, and R is the ratio of heavy to light isotope of carbon or nitrogen.

Reference materials used were internal standards of known relationship to the international standards of Vienna Pee Dee belemnite for carbon isotopes and atmospheric nitrogen for nitrogen isotopes. Stable isotope ratios are expressed as parts per thousand (‰) delta values relative to the international standards for carbon and nitrogen. White muscle tissue of northern pike (*Esox lucius* L.) (for animal based samples) and birch leaves (*Betula pendula* L.) (for detritus, macrophytes and periphyton) with known isotopic compositions were used as internal working standards to ensure precision of the analyses. One standard sample was run repeatedly after every six samples in each sequence. Standard deviations within reference samples in each sequence were less than 0.1‰ for carbon and 0.2‰ for nitrogen in pike and in birch leaf samples.

### Stomach content analysis

To provide further information about diet of the signal crayfish, the same crayfish analyzed for isotopes were dissected, and their foreguts were removed. Contents of each foregut were placed in a Petri dish containing a small amount of water and were analyzed using a dissecting microscope (50×). Food items were identified and divided into macroinvertebrates, vertebrates (fish parts), crayfish parts, periphyton, and detritus (CPOM). Identification of macroinvertebrates were based on sclerotized body parts, particularly head capsules, mouth parts, and leg fragments^55^. Abundance of each food item was estimated by sight and divided in four classes of abundance: 0 = 0–25%, 1 = 25–50%, 2 = 50–75%, 3 = 75–100%^56^. Percentage of occurrence (%Oi) of food items in adults and juveniles in summer and autumn were calculated as:

2 $$\%Oi = (Ji / P)\times 100$$

where J_i_ is the number of crayfish containing prey *i*, and *P* is the number of crayfish with food in their stomach.

### Trophic niches and food sources contribution

Trophic niche widths of signal crayfish adults and juveniles in summer and autumn were determined using the SIBER-package (Stable Isotope Bayesian Ellipses in R)^57^ in R^58^, which takes into account different numbers of samples^47^. Trophic niche similarity between crayfish adults and juveniles in different seasons was also quantified by calculating niche overlap as a proportion of the non-overlapping area of the two trophic niche ellipses^59^. The proportion range varies between 0 and 1 depending on if ellipses are completely distinct or completely overlapping^59^.

Bayesian mixing models MixSIAR^60,61^ were used for determining seasonal differences in food source usage between the two signal crayfish life stages and sexes. Models were run separately for summer and autumn for adults and juveniles using carbon and nitrogen stable isotope values from signal crayfish individuals and three potential food sources (detritus, periphyton and macroinvertebrates). Crayfish was included as a food source only for adult crayfish. MixSIAR includes different covariates as random and fixed effects, continuous variables, and different model error combinations as process * residual, allowing more robust results^59,60^. In this study, models were run with age classes and sex employed as fixed effects and selecting residual and process errors^62^. We used general fractionation factors for the aquatic organisms collected from the literature as no reliable signal crayfish specific values were available. Some authors e.g.^63–65^ have examined crayfish specific fractionation factors but often experiments crayfish were fed on either single plant or meat diet in both carbon and nitrogen could vary a lot. Instead, general values with wide range (SD) should give feasible results in mixing models. Food source fractionation factors were assumed as 3.23 ± 0.41‰ for δ^15^N and 0.47 ± 1.23‰ for δ^13^C for macroinvertebrates and crayfish, respectively^66^, and 2.4 ± 0.42‰ for δ^15^N and 0.40 ± 0.28‰ for δ^13^C for detritus and periphyton, respectively^67^.

The models were run using Markov Chain Monte Carlo (MCMC) parameters of three chains of 300,000 iterations, burn-in phase of 200,000, and thinning of 100. Percentage contribution of food sources of signal crayfish diet were generated by the models as averages with 95% credibility intervals, according to sex and age classes, for each season. All model results were tested for convergence and diagnostic statistics using the Gelman-Rubin and Geweke tests. For the first test all variables must have values < 1.05 and for the second test means of the first and second part of the chain must be the same. All statistical analyses, including SIBER and MixSIAR models, were conducted in R^58^.

### Ethics declarations

We confirm that our manuscript is neither been submitted nor published elsewhere. Research was conducted in adherence to publishers Ethical Guidelines. All of the authors agree with the journal publication policy, including data publication policy, and reviewing all publication decisions procedure.

## Supplementary Information

Supplementary Table S1.

## Data Availability

The datasets generated during and/or analyzed during the current study are available from the corresponding author on reasonable request.
