# Publisher Correction: Diet and trophic niche of the invasive signal crayfish in the first invaded Italian stream ecosystem

**DOI:** 10.1038/s41598-021-00571-5

**Published:** 2021-10-26

**Authors:** Fabio Ercoli, Daniela Ghia, Laura Gruppuso, Gianluca Fea, Tiziano Bo, Timo J. Ruokonen

**Affiliations:** 1 Chair of Hydrobiology and Fishery, Institute of Agricultural and Environmental Sciences, Estonian University of Life Sciences, Kreutzwaldi 5D, 51006 Tartu, Estonia; 2 Department of Biological and Environmental Sciences, University of Jyväskylä, Survontie 9C, 40014 Jyväskylä, Finland; 3 Dipartimento di Scienze della Terra e dell’Ambiente, Università degli Studi di Pavia, Pavia, Italy; 4 Dipartimento di Scienze della Vita e Biologia dei Sistemi, Università degli Studi di Torino, Via Accademia Albertina 13, 10123 Turin, Italy; 5 NaturaStaff Hydrobiologist, Via Lunga, 14040 Mongardino, AT Italy; 6 Natural Resources Institute Finland, Survontie 9A, 40500 Jyväskylä, Finland

Correction to: *Scientific Reports* https://doi.org/10.1038/s41598-021-88073-2, published online 22 April 2021

The original version of this Article contained errors in Table 1 and Table 2.

In Table 1, the division lines between Sites (“S1”, “S2” and “S3”) and Age class (“Adults” and “Juveniles”) was not clear. Each Site had to contain the Age class “Adults” and “Mean”, and “Juveniles” and “Mean” values.

In Table 2, the division lines between Sites (“S1”, “S2” and “S3”) and Food sources was incorrectly placed. Each Site had to contain five Food sources: “Macroinvertebrates”, “Crayfish”, “Detritus”, “Periphyton” and “Macrophytes”.

The original Table 1 and Table 2 and accompanying legends appear below.

**Table 1:** Signal crayfish mean (± SD) stable isotope values of carbon and nitrogen, carapace length (CL), number of sampled individuals between sexes, age classes, and sites in Valla Stream in summer and autumn.

Seasons	Sites	Age class	Sex	N	CL	δ^13^C	δ^15^N
Summer		Adults	M	7	47.17 ± 14.3	− 26.38 ± 0.84	4.63 ± 0.27
			F	5	39.63 ± 10.3	− 26.53 ± 0.50	4.73 ± 0.36
	S1	Mean values			44.03 ± 13.3	− 26.44 ± 0.72	4.67 ± 0.32
		Juveniles	M	5	26.41 ± 2.6	− 26.16 ± 0.30	4.89 ± 0.12
			F	5	25.95 ± 2.6	− 26.28 ± 0.43	4.42 ± 0.21
		Mean			26.18 ± 2.6	− 26.22 ± 0.38	4.65 ± 0.29
		Adults	M	9	49.53 ± 12.0	− 26.58 ± 0.70	4.91 ± 0.62
			F	10	47.34 ± 7.3	− 26.67 ± 0.61	5.00 ± 0.34
	S2	Mean			48.38 ± 9.9	− 26.62 ± 0.66	4.96 ± 0.50
		Juveniles	M	8	25.39 ± 2.9	− 26.28 ± 0.43	5.03 ± 0.56
			F	12	21.58 ± 7.5	− 26.36 ± 0.56	4.95 ± 0.64
		Mean			23.10 ± 6.4	− 26.33 ± 0.51	4.98 ± 0.61
		Adults	M	13	43.03 ± 9.0	− 27.08 ± 0.80	6.25 ± 1.60
			F	12	41.63 ± 5.2	− 26.42 ± 0.44	5.13 ± 0.99
	S3	Mean			42.36 ± 7.5	− 26.76 ± 0.73	5.71 ± 1.46
		Juveniles	M	7	26.21 ± 2.5	− 26.53 ± 0.37	6.60 ± 1.80
			F	11	21.28 ± 8.8	− 26.10 ± 0.82	6.72 ± 1.86
		Mean			23.20 ± 7.5	− 26.27 ± 0.71	6.67 ± 1.84
Autumn		Adults	M	3	48.97 ± 8.18	− 27.10 ± 0.88	5.03 ± 0.26
			F	3	39.38 ± 3.16	− 26.41 ± 0.21	4.69 ± 0.38
	S1	Mean			44.18 ± 7.84	− 26.76 ± 0.72	4.86 ± 0.37
		Juveniles	M	1	26.57	− 25.95	4.36
			F	4	19.26 ± 3.78	− 25.30 ± 0.09	4.56 ± 0.43
		Mean			20.72 ± 4.47	− 25.43 ± 0.27	4.52 ± 0.40
		Adults	M	4	37.80 ± 4.32	− 26.77 ± 0.29	4.77 ± 0.40
			F	–	–	–	–
	S2	Mean			37.80 ± 4.32	− 26.77 ± 0.29	4.77 ± 0.40
		Juveniles	M	3	25.76 ± 1.42	− 26.38 ± 0.28	4.75 ± 0.53
			F	10	25.29 ± 2.77	− 26.86 ± 0.29	4.85 ± 0.61
		Mean			25.40 ± 2.53	− 26.75 ± 0.34	4.82 ± 0.58
		Adults	M	4	51.38 ± 9.89	− 26.86 ± 0.70	5.97 ± 1.21
			F	2	44.24 ± 11.57	− 26.50 ± 0.11	6.05 ± 1.19
	S3	Mean			49.00 ± 11.01	− 26.74 ± 0.53	5.99 ± 0.99
		Juveniles	M	4	24.29 ± 5.34	− 26.11 ± 0.49	6.55 ± 0.85
			F	5	22.97 ± 6.65	− 26.28 ± 0.42	6.83 ± 1.03
		Mean			23.56 ± 6.14	− 26.21 ± 0.40	6.71 ± 0.86

**Table 2:** Mean (± SD) stable isotope values of carbon and nitrogen of food sources at each site in Valla Stream in summer and autumn.

Sites	Food sources	Summer			Autumn		
		N	δ^13^C	δ^15^N	N	δ^13^C	δ^15^N
	Macroinvertebrates	3	− 26.74 ± 1.11	3.08 ± 1.28	3	− 29.53 ± 2.33	2.31 ± 0.99
S1	Crayfish	–	− 26.34 ± 0.60	4.66 ± 0.30	–	− 26.15 ± 0.86	4.71 ± 0.41
	Detritus	3	− 28.01 ± 0.05	− 1.57 ± 0.10	3	− 30.01 ± 0.62	− 2.44 ± 0.63
	Periphyton	–	− 26.88 ± 1.99	1.91 ± 1.46	3	− 27.32 ± 0.30	0.88 ± 0.12
	Macrophytes	3	− 39.61 ± 0.20	1.92 ± 0.35	3	− 39.20 ± 0.10	0.86 ± 0.16
	Macroinvertebrates	3	− 27.92 ± 1.89	2.60 ± 1.30	3	− 30.41 ± 2.88	2.72 ± 0.82
S2	Crayfish	–	− 26.47 ± 0.60	4.97 ± 0.55	–	− 26.76 ± 0.32	4.81 ± 0.54
	Detritus	3	− 28.92 ± 0.36	− 2.07 ± 0.03	3	− 29.06 ± 0.46	− 2.62 ± 0.24
	Periphyton	–	− 26.88 ± 1.99	1.91 ± 1.46	3	− 28.24 ± 2.16	1.89 ± 1.38
	Macrophytes	3	− 40.52 ± 0.72	0.42 ± 0.17	3	− 39.59 ± 0.08	1.10 ± 0.21
	Macroinvertebrates	3	− 27.83 ± 1.34	3.95 ± 2.24	3	− 29.52 ± 1.65	3.77 ± 1.08
S3	Crayfish	–	− 26.55 ± 0.76	6.11 ± 1.69	–	− 26.42 ± 0.52	6.42 ± 0.97
	Detritus	3	− 29.18 ± 0.38	− 1.53 ± 0.31	3	− 29.76 ± 0.73	− 2.15 ± 0.31
	Periphyton	–	− 26.88 ± 1.99	1.91 ± 1.46	3	− 24.26 ± 0.74	2.65 ± 0.45
	Macrophytes	3	− 39.15 ± 0.49	0.34 ± 0.038	3	− 38.85 ± 0.03	0.85 ± 0.18

The original Article has been corrected.

