# Author Correction: Diet and trophic niche of the invasive signal crayfish in the first invaded Italian stream ecosystem

**DOI:** 10.1038/s41598-023-29693-8

**Published:** 2023-02-14

**Authors:** Fabio Ercoli, Daniela Ghia, Laura Gruppuso, Gianluca Fea, Tiziano Bo, Timo J. Ruokonen

**Affiliations:** 1 Chair of Hydrobiology and Fishery, Institute of Agricultural and Environmental Sciences, Estonian University of Life Sciences, Kreutzwaldi 5D, 51006 Tartu, Estonia; 2 Department of Biological and Environmental Sciences, University of Jyväskylä, Survontie 9C, 40014 Jyväskylä, Finland; 3 Dipartimento di Scienze della Terra e dell’Ambiente, Università degli Studi di Pavia, Pavia, Italy; 4 Dipartimento di Scienze della Vita e Biologia dei Sistemi, Università degli Studi di Torino, Via Accademia Albertina 13, 10123 Turin, Italy; 5 NaturaStaff Hydrobiologist, Via Lunga, 14040 Mongardino, AT Italy; 6 Natural Resources Institute Finland, Survontie 9A, 40500 Jyväskylä, Finland

Correction to: *Scientific Reports* 10.1038/s41598-021-88073-2, published online 22 April 2021

The Acknowledgements section in the original version of this Article was incomplete.

“This study was supported by the Estonian Research Council, Mobilitas Pluss research project (MOBJD29 to Fabio Ercoli), the Estonian Ministry of Education and Research (institutional research funding projects IUT 21-2 to Tiina Nõges), the Estonian University of Life Sciences (research project P190254PKKH to Fabio Ercoli) and the European Union H2020 WIDESPREAD (TREICLAKE 951963). Thanks to Dr. Mark J. McCarthy for revising the English of the manuscript.”

now reads:

“This study was supported by the Estonian Research Council, Mobilitas Pluss research project (MOBJD29 to Fabio Ercoli), the Estonian Ministry of Education and Research (institutional research funding projects IUT 21-2 to Tiina Nõges), the Estonian University of Life Sciences (research project P190254PKKH to Fabio Ercoli) and the European Union H2020 WIDESPREAD (TREICLAKE 951963). This project has received funding from the European Union’s Horizon 2020 research and innovation programme under grant agreement No 951963. Thanks to Dr. Mark J. McCarthy for revising the English of the manuscript."

The original Article has been corrected.

