# Supplementary material for: Diet and trophic niche of the invasive signal crayfish in the first invaded Italian stream ecosystem
Source: Sci Rep. 2021 Apr 22;11:8704. doi: 10.1038/s41598-021-88073-2 (PMC8062500; doi:10.1038/s41598-021-88073-2)
Supplement: Supplementary file 1 — Supplementary Table S1. [file 41598_2021_88073_MOESM1_ESM.pdf]

**Diet and trophic niche of the invasive signal crayfish in the first invaded Italian stream ecosystem**

Fabio Ercoli[1,2]*, Daniela Ghia[3], Laura Gruppuso[4], Gianluca Fea[3], Tiziano Bo[5], Timo J. Ruokonen[2,6]

[1]Estonian University of Life Sciences, Institute of Agricultural and Environmental Sciences, Chair of Hydrobiology and Fishery, Kreutswaldi 5, 51006, Tartu, Estonia

[2]University of Jyväskylä, Department of Biological and Environmental Sciences, Survontie 9C, 40014, Jyväskylä, Finland

[3]Università degli Studi di Pavia, Dipartimento di Scienze della Terra e dell'Ambiente, Pavia, Italy

[4]Università degli Studi di Torino, Dipartimento di Scienze della Vita e Biologia dei Sistemi, Via Accademia Albertina 13, 10123 Turin, Italy

[5]NaturaStaff Hydrobiologist, Via Lunga, 14040 Mongardino (AT), Italy

[6]Natural Resources Institute Finland, Survontie 9 A, 40500, Jyväskylä, Finland

*Corresponding author email and ORCID: fabio.ercoli@emu.ee

0000-0003-2433-6222

Table S1. Mean values (± SD) of signal crayfish catch per unit effort (CPUE) from all sites studied in Stream Valla from April 2015 to March 2016. CPUE represents the number of crayfish caught per trap per number of trapping nights.

| Month-year | CPUE |
|---|---|
| Apr-15 | 0.35±0.17 |
| May-15 | 0.55±0.32 |
| Jun-15 | 2.17±1.53 |
| Jul-15 | 4.55±1.32 |
| Aug-15 | 2.98±1.51 |
| Sep-15 | 1.67±1.04 |
| Oct-15 | 1.47±1.24 |
| Nov-15 | 1.38±1.09 |
| Dec-15 | 0.65±0.52 |
| Jan-16 | 0.23±0.23 |
| Feb-16 | 0.33±0.47 |
| Mar-16 | 0.03±0.04 |
